# Vitamin D supplementation worsens Alzheimer's progression: Animal model and human cohort studies

**DOI:** 10.1111/acel.13670

**Published:** 2022-07-12

**Authors:** Rai‐Hua Lai, Chih‐Cheng Hsu, Ben‐Hui Yu, Yu‐Ru Lo, Yueh‐Ying Hsu, Mei‐Hsin Chen, Jyh‐Lyh Juang

**Affiliations:** ^1^ Institute of Molecular and Genomic Medicine National Health Research Institutes Miaoli Taiwan; ^2^ National Center for Geriatrics and Welfare Research National Health Research Institutes Miaoli Taiwan; ^3^ Institute of Population Health Sciences National Health Research Institutes Miaoli Taiwan; ^4^ Department of Health Services Administration China Medical University Taichung Taiwan; ^5^ Department of Family Medicine Min‐Sheng General Hospital Taoyuan Taiwan; ^6^ Ph.D. Program for Aging China Medical University Taichung Taiwan

**Keywords:** Alzheimer's disease, longitudinal study, non‐genomic vitamin D signaling, p53, vitamin D, vitamin D receptor

## Abstract

Vitamin D deficiency has been epidemiologically linked to Alzheimer's disease (AD) and other dementias, but no interventional studies have proved causality. Our previous work revealed that the genomic vitamin D receptor (VDR) is already converted into a non‐genomic signaling pathway by forming a complex with p53 in the AD brain. Here, we extend our previous work to assess whether it is beneficial to supplement AD mice and humans with vitamin D. Intriguingly, we first observed that APP/PS1 mice fed a vitamin D‐sufficient diet showed significantly lower levels of serum vitamin D, suggesting its deficiency may be a consequence not a cause of AD. Moreover, supplementation of vitamin D led to increased Aβ deposition and exacerbated AD. Mechanistically, vitamin D supplementation did not rescue the genomic VDR/RXR complex but instead enhanced the non‐genomic VDR/p53 complex in AD brains. Consistently, our population‐based longitudinal study also showed that dementia‐free older adults (*n* = 14,648) taking vitamin D_3_ supplements for over 146 days/year were 1.8 times more likely to develop dementia than those not taking the supplements. Among those with pre‐existing dementia (*n* = 980), those taking vitamin D_3_ supplements for over 146 days/year had 2.17 times the risk of mortality than those not taking the supplements. Collectively, these animal model and human cohort studies caution against prolonged use of vitamin D by AD patients.

## Graphical abstract

We studied whether it is beneficial to supplement vitamin D to APP/PS1 mice and humans. The animal studies show vitamin D deficiency is an early disease outcome and supplementation of vitamin D worsens AD brain pathology via enhancing VDR/p53 complex to impair autophagosome. Nationwide longitudinal cohort studies also suggest long‐term vitamin D supplementation is associated with higher risk of dementia in dementia‐free older adults and higher mortality in dementia individuals. Our results caution against prolonged use of vitamin D by AD patients and older adults.

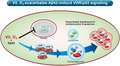

## 1 INTRODUCTION

Alzheimer's disease (AD) and other neurodegenerative dementia diseases are rapidly becoming a public health issue (World Health Organization, 2020). Current medications only have a minimal beneficial effect on these diseases, so older people are being asked to take preventive measures against dementia. While AD is a multifarious disease and its underlying mechanisms have not been clearly defined, it is recommended that older people get long‐term regular exercise and lead healthy lifestyles to reduce the risk of dementia (Wittfeld et al., 2020). However, these recommendations require people to change long‐established unhealthy behaviors, which some people can change but many cannot change over the long term.

Dietary supplementation has also been suggested (Allen et al., 2013; Jennings et al., 2020). Many recent epidemiological studies have found a link between vitamin D deficiency and risk of dementia. Basing their assumptions on results of observational studies (Shen & Ji, 2015; Sommer et al., 2017), some people have come to believe that vitamin D deficiency causes AD and have gone a step further to conclude that vitamin D supplementation might confer neuroprotection against dementia (Banerjee et al., 2015; Landel, Annweiler, et al., 2016a). After all, one disease, rickets, has already been found to be caused by vitamin D and/or calcium deficiency in infants and children and has been found to be prevented with supplemental vitamin D (Sahay & Sahay, 2012). In contrast to the beneficial effect on bone development seen in developing children, results of randomized clinical trials in adults have found no overall health benefit for supplementation of vitamin D (Barbarawi et al., 2019; Jackson et al., 2006). Nevertheless, some still believe that a safe optimal dosage of vitamin D (1000–2000 IU) can be used daily to achieve an adequate serum vitamin D level without any significant side effects (Bischoff‐Ferrari et al., 2006; Gorham et al., 2007; Vieth et al., 2007).

One concern that is often ignored by those recommending supplementation with vitamin D is that it is actually a steroid hormone and prolonged use of it in AD patients or older people is poorly understood (Anastasiou et al., 2014). Another important concern is raised from the results of our previous work that suggest the genomic vitamin D signaling pathway is already impaired in AD brain (Lai et al., 2021). However, no large‐scale population study has been conducted to investigate what effect prolonged supplementation of vitamin D might have on AD. In the present study, we assumed that vitamin D supplementation started at a very early disease stage in AD mice cannot represent real‐world conditions in humans (Landel, Millet, et al., 2016b; Yu et al., 2011). Therefore, we performed a very similar animal experiment, but starting the supplementation at mid‐stage disease in mice, when vitamin D deficiency is evident, to assess the potential impact of vitamin D supplementation on the pathogenesis of AD. We also performed a population‐based longitudinal study to investigate whether the response to vitamin D supplementation in humans is similar to that in our experimental mouse model. The results of this work are of potential value in assessment of the outcomes of long‐term use of vitamin D supplementation in AD patients.

## 2 METHODS

### 2.1 Mice

Double transgenic APP/PS1 mice (Cat# 037565‐JAX, RRID: MMRRC_037565‐JAX) were purchased from Jackson Laboratory (Bar Harbor, ME, USA) and bred with wild‐type B6C3F1/Bltw (C57BL/6 N background) mice. The sample size of mouse studies was chosen following the guidelines of “Guidelines for care and uses of mammals in neuroscience and behavioral research” (Van Sluyters & Obernier, 2004). Mice were allocated randomly in all experiments. Mice of 4.5 months of age were fed on a normal diet (Altromin; Cat# Altromin 1320) containing sufficient vitamin D_3_ (600 IU/Kg of cholecalciferol) or D_3_‐supplemented diet (Match to Altromin 1320 with 8044 IU/Kg of cholecalciferol; Research Diets, Inc; Cat# D13031002) with or without intraperitoneally injected weekly with 3 mg/kg of p53 inhibitor Pifithrin‐α (PFTα, Sigma‐Aldrich, Cat# P4359) for 3 or 7.5 months before subsequent assays were conducted. Submandibular blood collection and cerebrospinal fluid (CSF) collection from anesthetized mice as described (Lim et al., 2018) were performed on APP/PS1 and wild‐type mice at the indicated time points for quantification of serum 25(OH) D_3_ levels by using Vitamin D_3_ EIA Kit (Cayman Chemical, Cat# 501050). The Morris water maze test were performed in NHRI Animal Behavior Core Facility following the procedures as described previously (Lai et al., 2021). Animal procedures and protocols were approved by the Institutional Animal Care and Use Committee at NHRI (approved protocol no. NHRI‐IACUC‐101057‐A and NHRI‐IACUC‐103136‐A).

### 2.2 Cell culture, cell viability, TUNEL staining and mammalian two‐hybrid assay

SH‐SY5Y (human neuroblastoma, ATCC CRL‐2266) cells were cultured as described (Lai et al., 2021). The cell viability was treatment with or without calcitriol (Sigma‐Aldrich, Cat# D1530, 1,25(OH)_2_D_3_) or calcidiol (Sigma‐Aldrich, Cat# 17938, 25(OH)D_3_) and measured by colorimetric WST‐1 assay. The TUNEL assay was performed by using ApoAlert™ DNA Fragmentation Assay Kit (Clontech, Cat# 630107). Mammalian two‐hybrid assay was used to assess the interaction between VDR and RXR by using Dual‐Luciferase Reporter Assay (Promega, Cat# E1910) for SH‐SY5Y cells with pretreatment with Aβ42 (1–10 μM) followed by treatment with or without calcitriol for 6 h before the assay.

### 2.3 Antibody

The antibodies used in this study are listed as follows: VDR (C20), Santa Cruz Biotechnology, Cat# sc‐1008; VDR(D6), Santa Cruz, Biotechnology Cat# sc‐1313; GAPDH, GeneTex, Cat# GTX10011; PARP‐1/2 (H‐250), Santa Cruz Biotechnology, Cat# sc‐715; LC3B, Cell Signaling Technology, Cat# 4108; Beta‐Amyloid‐1‐16 antibody, BioLegend, Cat# 803014; β‐Amyloid Antibody, Cell Signaling Technology, Cat# 245; BACE (M‐83), Santa Cruz Biotechnology, Cat# sc‐10748; GFAP (Clone SP78), MybioSourse, Cat# MBS302899; GFAP (GA5), Cell Signaling Technology, Cat# 3670; p53 (DO‐1) Santa Cruz Biotechnology Cat# sc‐12; Cat# sc‐13985; Phospho‐SQSTM1/p62 (Ser349), Cell Signaling Technology, Cat# 95697; SQATM1/p62 (GT1478), Thermo Fisher Scientific, Cat# MA5–27800; TNF‐α (D2D4) XP® Rabbit mAb, Cell Signaling Technology, Cat# 1194; Alexa 488 chicken anti‐rabbit IgG (H + L), Thermo Fisher Scientific, Cat# A‐21441; Alexa 594 chicken anti‐goat IgG (H + L), Thermo Fisher Scientific Cat# A‐2146; Peroxidase‐AffiniPure Goat Anti‐Rabbit IgG (H + L), Jackson ImmunoResearch Labs, Cat# 111‐035‐144; Peroxidase‐AffiniPure Goat Anti‐Mouse IgG (H + L), Jackson ImmunoResearch Labs, Cat# 115‐035‐14; Peroxidase‐AffiniPure Rabbit Anti‐Goat IgG (H + L), Jackson ImmunoResearch Labs, Cat# 305‐035‐003; Mouse anti‐Rabbit light chain; HRP conjugate, Millipore, at# MAB201P; HRP‐conjugated AffiniPure Mouse Anti‐Rabbit IgG Light Chain, Bclonal Cat# AS061.

### 2.4 Population‐based study

#### 2.4.1 Study design and data sources

We conducted a retrospective population‐based cohort study aiming at delineating the effects of calcitriol use on dementia development in the dementia‐free older adults and the mortality impact on subjects with dementia. There was about 99% of Taiwan's population of 23 million enrolled in the National Health Insurance (NHI) program since 1995. The Taiwan NHI Research Database (NHIRD), derived from the reimbursement claims within the NHI program, provides detailed medical utilization information of the NHI beneficiaries. The NHIRD was the data source we used to select calcitriol users for this study.

The Longitudinal Health Insurance Database for the year 2000 (LHID2000), one of the data components in NHIRD, consists of claims information of one million subjects who were randomly selected from the 2000 Registry of Beneficiaries of the NHI program. The LHID2000 served as the sampling pool from which we selected the calcitriol nonusers for the first analysis.

The Registry of Catastrophic Illness Patients (RCIP) is another dataset of the NHIRD, containing information of NHI utilization of all patients with catastrophic illnesses defined by the Taiwan's government. A rigorous clinical review and evaluation would precede entitlement for the RCIP to assure valid diagnoses. The catastrophic injuries/illnesses in Taiwan included 31 categories of major illnesses (e.g., cancer, dialysis, hemophilia, etc.), with which patients are exempt from co‐payment and may thus avoid financial hardship. Dementia (ICD9 290 or 294) is one of the listed catastrophic illnesses. The RCIP is another dataset we used to conduct the second analysis.

#### 2.4.2 Analysis of the risk of incident dementia

From the data source of NHIRD, we identified those who were 65 years or older, free of dementia diagnosis before 2000, and had received at least one calcitriol prescription in 2000–2009 (*n* = 20,108) as the calcitriol users (Figure 3a). To exclude subjects less adherent to the study medication, we excluded the users with very short‐term calcitriol use (less than ten 2.5 mcg capsules per year) and the users who were diagnosed with dementia within 6 months after the first prescription of calcitriol had been made. From the LHID2000, we considered subjects as the calcitriol nonusers if they were 65 years of age or older, free of dementia diagnosis before 2000, and never had calcitriol prescription in 2000–2010 (*n* = 89766). To increase comparability, the calcitriol users were propensity score‐matched to their nonuser counterparts on a 1:1 ratio based on age (birthday year), gender, calcium prescription (yes/no), and comorbidities (yes/no) including chronic kidney disease (ICD9: 582, 585, 586, 583.0, 583.1, 583.2, 583.3, 583.4, 583.5, 583.6, 583.7, A‐code: A350), osteoporosis (ICD9: 733.0 V17.81 V82.81, A‐code: A439 AV09 AV05), acquired hypothyroidism (ICD9:240–246, A‐code: A180), Diabetes (ICD9: 250, A‐code: A181), hyperlipidemia (ICD9: 272, A‐code: A182), hypertension (ICD9: 401 402 403 404 405, A‐code: A269 A260), and the Charlson's index score. After matching procedures, we got equal number of calcitriol users and nonusers (*n* = 7324) for further analysis. The subgroup analysis was conducted with the gender of male (*n* = 5362) and female (*n* = 9286), or the age of 65–75 years old (*n* = 5196) and older than 75 years old (*n* = 9452).

#### 2.4.3 Analysis of dementia mortality

From the RCIP in 2000–2009, we identified 51,606 individuals who were 60 years or older and had a diagnosis of dementia (ICD9 290 or 294), of whom 636 subjects had their first calcitriol prescription after the diagnosis and the remaining 50,970 subjects never on calcitriol were considered as the calcitriol nonusers (Figure 4a). To exclude subjects less adherent to the study medication, we excluded the users with very short‐term calcitriol use (less than ten 2.5 mcg capsules per year) and the users who died within 3 months after the first calcitriol prescription. To increase comparability, we also matched the calcitriol users to the nonusers on a 1:4 ratio based on the same covariates as described in the analysis of risk of incident dementia. Frequency matching was performed to ensure a similar distribution of the variables in each group. Baseline characteristics showed no difference between the two groups. After matching procedures, we kept 196 dementia patients who were also the calcitriol users and 784 dementia patients without taking calcitriol (the nonusers) for further analysis. The subgroup analysis was conducted with the gender of male (*n* = 315) and female (*n* = 665), or the age of 60–75 years old (*n* = 239) and older than 75 years old (*n* = 741).

#### 2.4.4 Study outcome

The study outcome in the analysis for the risk of incident dementia was designated to be dementia development, which was defined as the first diagnosis of dementia (ICD9: 290.0, 290.1, 290.2, 290.3, 290.4, 294.1, 331.0, 331.1, 331.2, A‐code: A210, A213, A22). For the analysis of the risk of dementia mortality, the study outcome was all‐cause mortality.

#### 2.4.5 Statistical analysis

We used chi‐square test and *t*‐test to detect the differences for categorical and continuous variables, respectively. Associations between calcitriol use and study outcomes were analyzed using Kaplan–Meier survival curves and log‐rank tests. We used Cox proportional hazard models to assess adjusted effects of calcitriol use on the study outcomes. The proportional hazards assumption was ascertained by comparing log–log survival curves for all time‐independent covariates. All assessed log–log survival plots indicated no violation of the assumption. Hazard ratios (HR) with 95% confidence interval (CI) were used to measure the risk of dementia or mortality. For calcitriol users, study entry was defined as the date of first calcitriol use; while for the nonusers, study entry was assigned as the same date as their matched user counterparts. In analysis 1, the outcome was dementia development and observations were censored on last clinical visits by 31 December 2010. In analysis 2, the outcome was death defined as withdrawal from the national health insurance registry and sustained lack of healthcare utilization thereafter for at least 1 year. The observation was censored on the date of the last clinic visit by 31 December 2010. A two‐tailed p value less than 0.005 was considered significant. We conducted analyses using SAS version 9.4.

## 3 RESULTS

### 3.1 APP/PS1 AD mice given vitamin D‐sufficient diet exhibit decreased serum vitamin D levels

**FIGURE 1:**
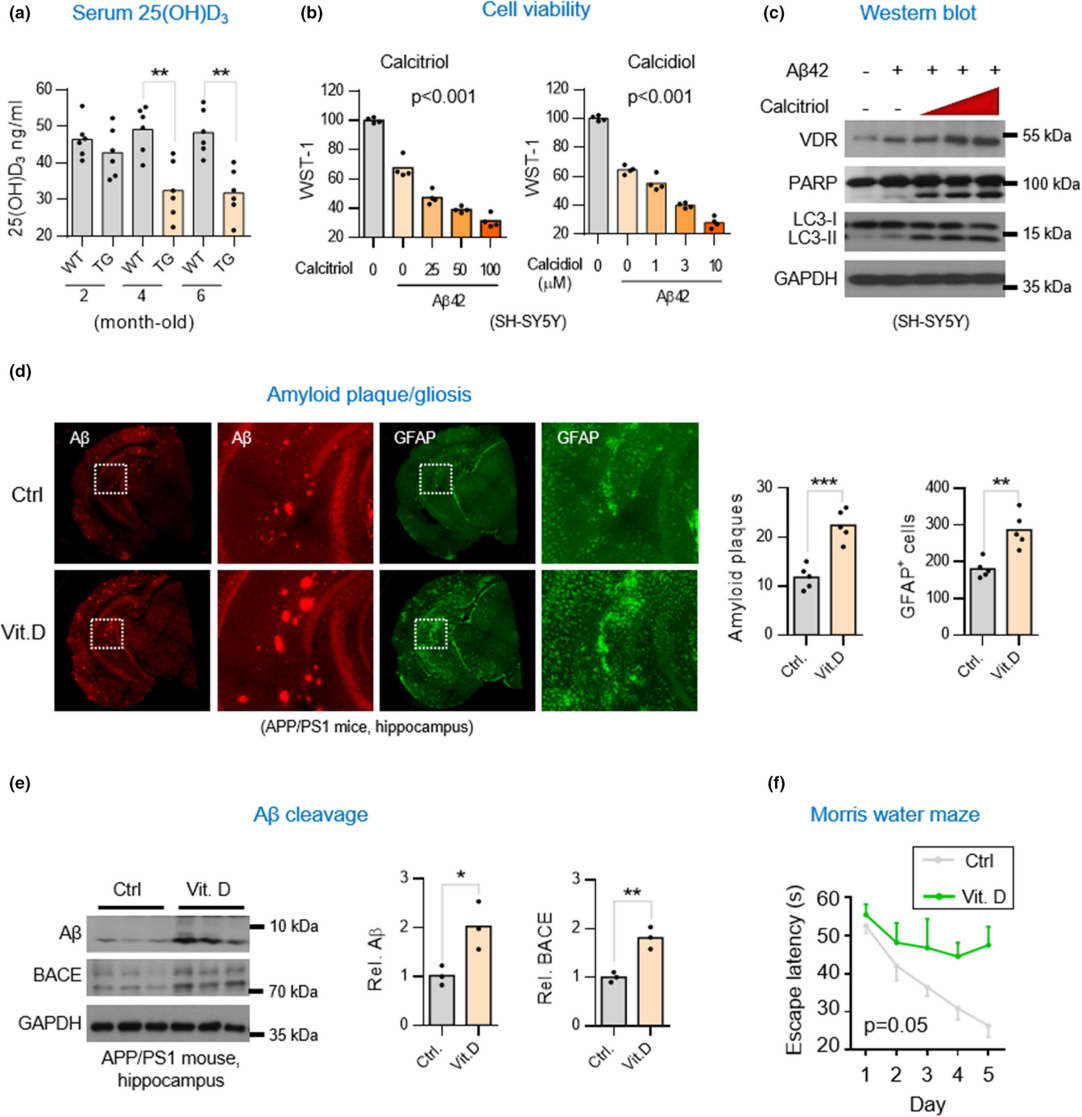
Dietary supplementation of vitamin D_3_ aggravates AD pathology in APP/PS1 AD mice. (a) Serum 25(OH)D_3_ levels in APP/PS1 (TG) and wild‐type (WT) mice. Mice were weaned at 4‐weeks of age (±3 days) and maintained on a vitamin D_3_‐sufficient diet (600 IU/Kg of cholecalciferol). Serum vitamin D_3_ levels were determined by 25(OH)D_3_ enzyme‐linked immunosorbent assay (EMSA) at the indicated time points (*n* = 5). Results are shown as mean ± SD. *p < 0.05; **p < 0.01; ***p < 0.001 by unpaired *t*‐test. (b) WST‐1 cell viability assay. SH‐SY5Y cells were exposed to vitamin D_3_ alone or Αβ (4 μM) plus vitamin D_3_ (calcitriol or calcidiol) for 6 h prior to assays. Results are shown as mean ± SD. *p < 0.05 by One‐way ANOVA. (c) Western blot analysis of VDR, apoptotic and autophagic marker proteins in SH‐SY5Y cells exposed to Aβ42 or plus without or without Vitamin D_3_. SH‐SY5Y cells were treated with 4 μM Aβ42 alone or Aβ42 plus 10, 30, or 100 nM calcitriol for 6 h before harvesting cell lysates for analysis. (d) Representative immunofluorescent micrographs of gliosis (anti‐GFAP, GA5) and amyloid aggregates (anti‐Αβ, D54D2) in hippocampal tissues of APP/PS1 mice. 4.5‐month‐old APP/PS1 mice were fed with vitamin D_3_‐supplemented (8044 IU/Kg cholecalciferol/day, Vit. D) or vitamin D_3_‐sufficient diets (600 IU/Kg cholecalciferol/day, Ctrl) for 3 months before harvesting brain tissues for analysis. Sections of cortex or hippocampus were stained with the indicated antibodies. The average percentage of surface area with Αβ plaques in five consecutive sections per animal (*n* = 4–7) was quantified by ImageJ in right panel. (e) Western blot analysis of Αβ production and β‐secretase 1 (BACE1) levels in hippocampal lysates of APP/PS1 mice supplemented with or without vitamin D_3._ Densitometrical quantification of Aβ and BACE bands were normalized to GAPDH (right panel). (f) Cognitive performance for AD mice supplemented with vitamin D_3_. 4.5‐month‐old APP/PS1 mice were fed with vitamin D_3_–fortified (Vit. D) or vitamin D_3_‐sufficient diets (Ctrl) for 7.5 months before Morris Water Maze. *p < 0.05 by One‐way ANOVA

Because vitamin D deficiency is linked to AD, it was imperative to clarify whether vitamin D deficiency is a risk factor or an outcome of AD. To test this hypothesis, we fed both APP/PS1 and wild‐type (WT) mice a vitamin D_3_‐sufficient diet (600 IU/Kg of cholecalciferol) and measured their serum vitamin D levels during the early stages of life. Intriguingly, beginning as early as 4 months, the AD mice started exhibiting significantly lower serum 25(OH)D_3_ levels compared with those 2 months old. There was no difference in serum vitamin D levels in WT over the study period (Figure 1a). We then tested whether the 25(OH)D_3_ levels were also decreased in the CSF of AD mice as seen in the serum. Indeed, the EIA assays showed that the 25(OH)D_3_ levels in CSF were also markedly decreased in AD mice compared with WT controls (Figure S1). These results may suggest that vitamin D deficiency may be caused by AD rather than caused by a lack of dietary vitamin D.

### 3.2 Faster disease progression after vitamin D supplementation in AD mice

Our previous finding that the VDR‐RXR heterodimer for transducing the genomic vitamin D signal was impaired in AD led us to question the seemingly common assumption that vitamin D supplementation may exert a protective effect on AD (Groves et al., 2014). We first assessed the potential impact of vitamin D supplementation on an Aβ42‐treated neuronal cell line. The results showed that the incubation of vitamin D (calcitriol or calcidiol) with SH‐SY5Y cells exposed to Aβ42 resulted in a significant dose‐dependent increase in apoptosis and autophagy (Figure 1b,c), suggesting that vitamin D might have a potentially damaging effect on neuronal cells exposed to Aβ42. Encouraged by this result, we proceeded to explore whether vitamin D supplementation could exert a similar detrimental effect on the progression of AD in mice. Adding 8044 IU/kg of cholecalciferol (vitamin D_3_) to the diets of APP/PS1 AD mice 4.5 months old for 3 months resulted in more severe Aβ plaque deposits and reactive gliosis in the hippocampus compared to controls (Figures 1d and S2). Western blot analyses also revealed increased levels of pro‐degenerative factors, including Aβ, β‐secretase 1 (BACE1), Nicastrin (a subunit of the γ‐secretase complex), TNF‐α, and autophagy in the hippocampal lysates of the experimental mice (Figures 1e and S2). Moreover, to exclude the possibility of a toxic dose effect in the supplementation of vitamin D, we examined the serum 25(OH)D_3_ levels and found that both the WT and AD mice maintained a physiological level of 25(OH)D_3_ after supplementation of 8,044 IU/kg calcidiol (Figure S2a).

Mice were then also subjected to the Morris water maze test. Mice administered vitamin D displayed worse cognitive functioning and performance behavior than the controls (Figure 1f). The results of these experiments suggest that over‐supplementation of vitamin D can exacerbate AD neurodegeneration.

### 3.3 Vitamin D supplementation enhances non‐genomic VDR/p53 signaling in worsening brain pathology in AD mice

**FIGURE 2:**
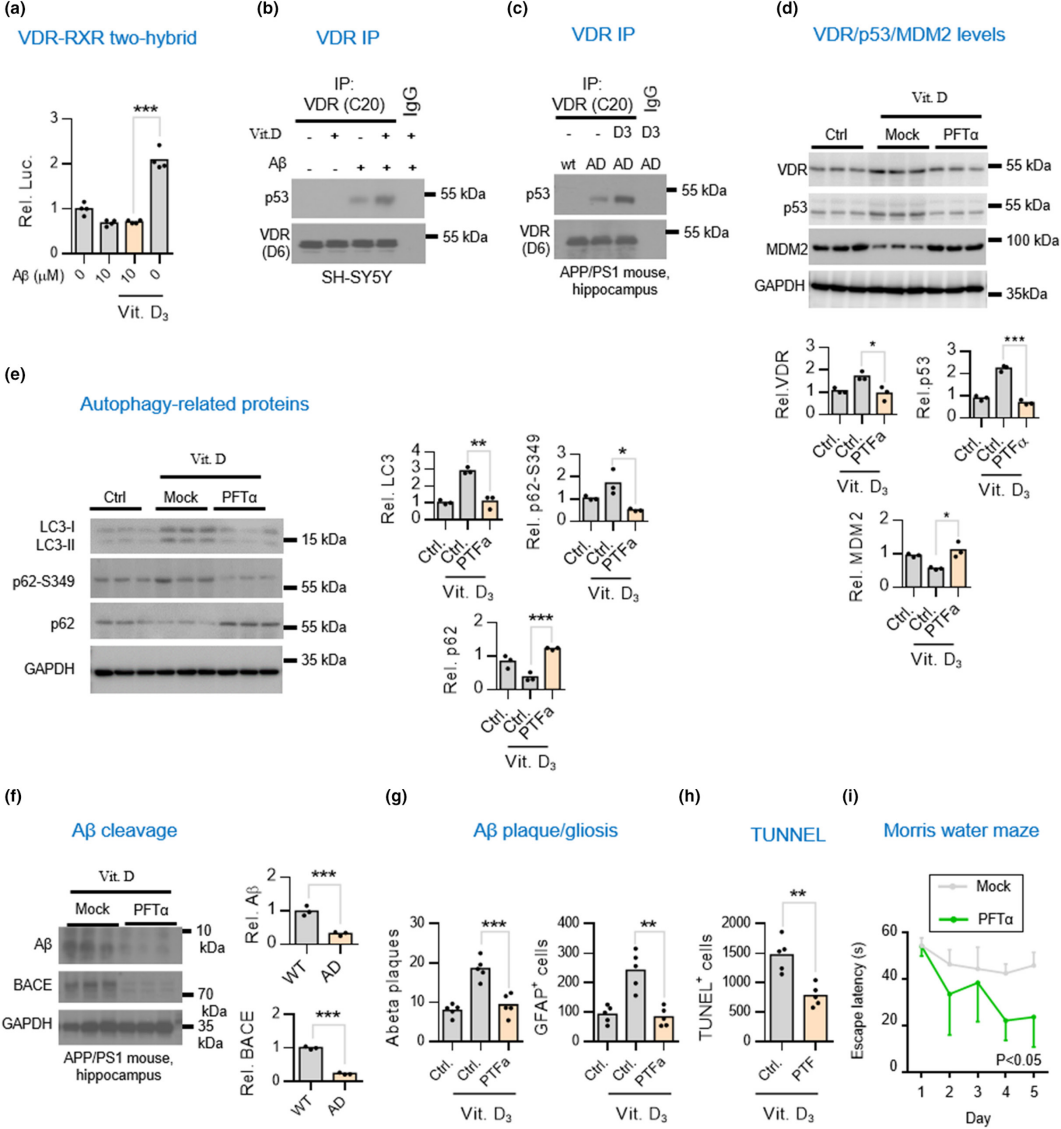
Vitamin D supplementation enhances VDR/p53 but not VDR/RXR complex in worsening brain pathology in APP/PS1 AD mice. (a) Mammalian two‐hybrid assays for studies of the interaction of VDR with RXR in neuronal cells exposed to Αβ plus with vitamin D_3_. SH‐SY5Y cells were treated Aβ42 for 6 h and then co‐treated with 10 nM calcitriol for additional 6 h prior to harvesting for mammalian two‐hybrid luciferase assays. (b,c) Western blot analysis of co‐immunoprecipitation of VDR/p53 complex in SH‐SY5Y cells and hippocampal tissues of APP/PS1 mice. (d) Western blot analysis of VDR, p53, and MDM2 in the hippocampal lysates of APP/PS1 mice treated with or without p53 inhibitor. 4.5‐month‐old APP/PS1 mice raised on vitamin D_3_‐sufficient diets were intraperitoneally injected weekly with 3 mg/kg of p53 inhibitor pifithrin‐α (PFTα) for 7.5 months before harvesting hippocampal tissues for analysis. Densitometrical quantification of VDR, p53, and MDM2 bands were normalized to GAPDH (lower panel). *p < 0.05; **p < 0.01; ***p < 0.001 by unpaired *t*‐test. (e) Western blot analysis of autophagic markers LC3, p62, and ser349 phosphorylated p62 (p62‐S349) in the hippocampal lysates of APP/PS1 mice injected with or without PFTα. Densitometrical quantification of LC3, p62‐S349, and p62 bands were normalized to GAPDH (right panel). (f) Western blot analysis of Αβ and BACE levels in the hippocampal lysates of APP/PS1 mice injected with or without PFTα. Densitometrical quantification of Aβ and BACE bands were normalized to GAPDH (right panel). (g,h) p53 inhibitor amelioration of vitamin D_3_‐aggravated Aβ aggregation and apoptosis. The Aβ, GFAP, and TUNEL‐positive signals in five consecutive sections per animal (*n* = 5) was quantified by ImageJ and presented as the mean ± SD. Scale bars, 50 μm. (i) Cognitive performance assays for the AD mice treated with p53 inhibitor. APP/PS1 mice were given with or without weekly injections of PTFα (*n* = 6 mice) starting at the age of 4.5‐month. APP/PS1 mice at 12‐month of age were used for the Morris Water Maze test

It is known that vitamin D normally induces VDR and RXR to combine, allowing the transduction of the genomic vitamin D signal (Yasmin et al., 2005). However, it is not known whether this occurs in the context of AD. To determine whether vitamin D would enhance the VDR/RXR complex formation in cells being exposed to Aβ42, we performed a biochemical study in which we added vitamin D_3_ to the SH‐SY5Y cells exposed to Aβ42. Surprisingly, vitamin D_3_ treatment did not enhance the VDR/RXR interaction in cell exposed to Aβ42 compared with cells exposed to vitamin D_3_ only (Figure 2a). This result suggested that the Aβ42 impairment of the VDR/RXR pathway was not rescued by vitamin D. Because our previous work had demonstrated that Aβ42 switched VDR binding partner from RXR to p53 to transduce the non‐genomic vitamin D signal in AD brain (Lai et al., 2021), we wanted to investigate whether the VDR/p53 complex formation was enhanced by vitamin D. We performed co‐immunoprecipitation assays and found that the Aβ‐triggered VDR/p53 complex was indeed further enhanced by the treatment of vitamin D_3_ (Figure 2b). Therefore, we continued to investigate whether vitamin D supplementation might also lead to a similar consequence enhancing VDR/p53 complex in AD mice. Indeed, after dietary supplementation of cholecalciferol for 3 months, APP/PS1 mice were found to have enhanced VDR/p53 interaction in the hippocampal tissues (Figure 2c). These results suggest vitamin D_3_ could aggravate Aβ42‐trigged VDR/p53 signaling promoting neuropathogenesis.

To further investigate whether the aggravation of VDR/p53 pathway by vitamin D would contribute to brain pathology in AD mice, we investigated whether we could reverse the adverse effects of vitamin D on AD by administering p53 inhibitor PFTα. Indeed, the p53 inhibitor PFTα not only markedly decreased protein levels of p53 and VDR but also increased the levels of MDM2, a protein that interacts with p53 promoting its degradation (Figure 2d). We also found that PFTα decreased their autophagic protein LC3II levels (Figure 2e). Because site‐specific phosphorylation of p62 has been implicated in the disruption of autophagy‐mediated protein degradation in AD brains, we also measured S349‐phosphorylated p62 (P‐S349) levels. We found p53 inhibitors had decreased P‐S349 levels, which are normally increased in the AD brain (Figure 2e, second panel).

Based on these findings, we believed we would also be able to observe improvement in brain lesions. Indeed, Western blot and immunohistochemistry studies both revealed significant attenuation in Aβ deposits, BACE activity, reactive gliosis, and neuronal apoptosis in the AD brains with vitamin D_3_ supplementation (Figure 2f–h). Finally, we wanted to know whether cognitive functioning and performance behavior would also be improved by treatment with a p53 inhibitor. Mice that were given the p53 inhibitor showed significant improvement in the Morris Water Maze test (Figure 2i). Taken together, these results suggest that Vitamin D_3_ supplementation aggravates VDR/p53 pathway in promoting brain pathology in APP/PS1 mice.

### 3.4 Vitamin D supplementation is associated with risk of dementia

**FIGURE 3:**
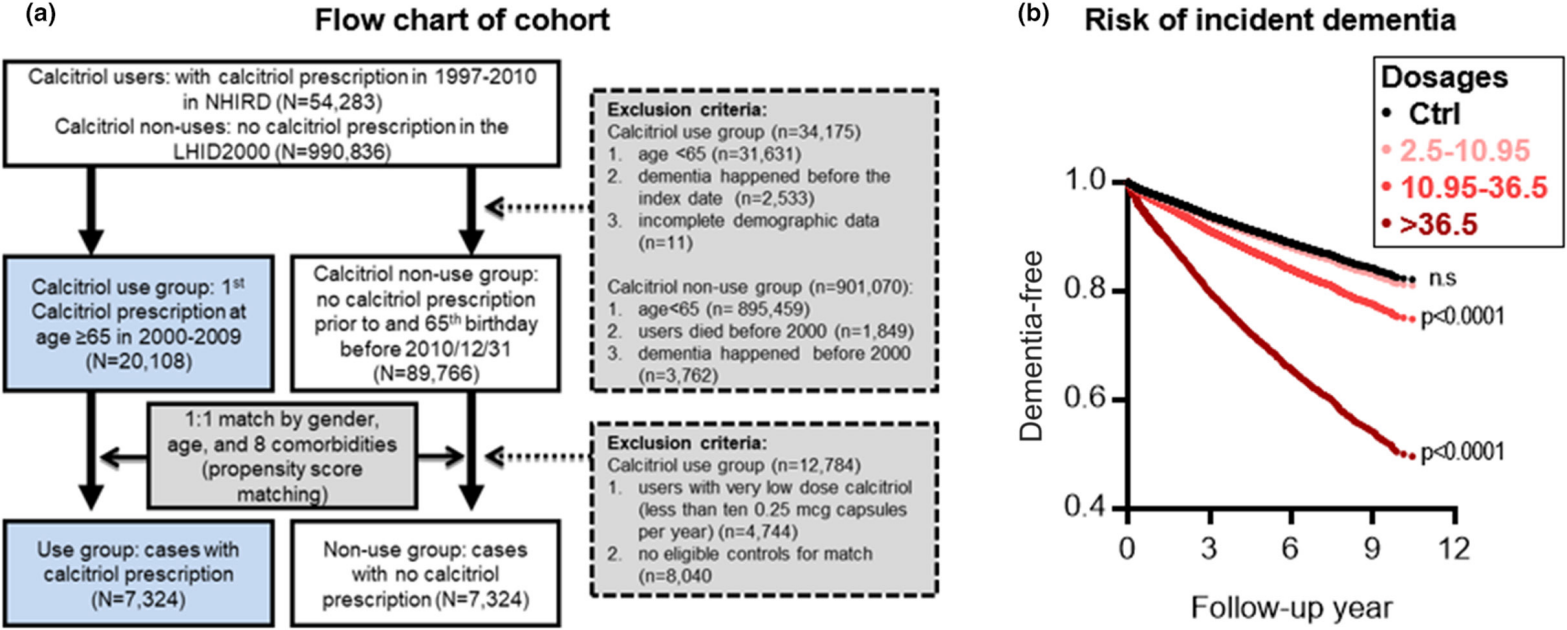
Population‐based cohort study of the associations between the incidence of dementia and calcitriol supplementation. (a) Flow chart of cohort formation for epidemiological study of association between incident dementia and calcitriol supplementation. NHIRD, National Health Insurance Research Database; LHID2000, Longitudinal Health Insurance Database 2000; Comorbidities including calcium prescription, chronic renal disease, osteoporosis, thyroid, diabetes, hyperlipidemia, hypertension, and Charlson score. (b) The adjusted curves of dementia development in study subjects aged over 65 years with different average cumulative dosages of calcitriol with a follow‐up of up to 10 years (*n* = 14,648). The definition of the ‘dosage/year’ is the assumed average maintenance dose (mcg) per year for calcitriol used in the whole follow‐up. ***p < 0.001 by Maximum Likelihood‐ratio test

Thus, we began to question whether vitamin D supplementation would, as has been suggested, be able to decrease the risk of AD. To find out, we performed a retrospective population‐based study to assess the effects of continuous use of an optimal dosage of calcitriol on the risk of dementia in older people (Figure 3a). We identified and enrolled 14,648 dementia‐free people aged over 65 years who had or had not been prescribed calcitriol from Taiwan's National Health Insurance Research Database (NHIRD) and then followed them for 11 years (2000–2010) to find out if they had received a diagnosis of incident dementia. Most claimants prescribed the vitamin took one tablet of calcitriol (0.25 mcg or 0.5 mcg) daily, the suggested optimal daily intake for this vitamin. Frequency matching was performed for age, gender, and eight comorbidities (prescriptions for calcium, chronic renal disease, osteoporosis, thyroid, diabetes, hyperlipidemia, hypertension, and Charlson score) and Cox proportional hazard model was used to assess the effects of calcitriol on incident dementia in three different dosage groups (Table 1). As can be seen in Figure 3b and Table S1, a low cumulative dose of calcitriol (<10.95 mcg/year or <43.8 capsules of 0.25 mcg per year) did not appear to affect the incident dementia. However, those taking a high (average >36.5 mcg/year or >146 capsules of 0.25 mcg per year) and medium (average 10.95–36.6 mcg/year or 43.8–146 capsules of 0.25 mcg per year) cumulative dose had a 1.80‐ and 1.27‐fold increase in risk of incident dementia, respectively, compared with nonusers, suggesting a potential link between prolonged use of calcitriol and increased risk of dementia in older people. Similar effects were also observed in the subgroup analysis of sex (Tables S2 and S3). When incidence of dementia was compared between sex, it was interesting to note that dementia‐free females appeared to be more sensitive than dementia‐free males to calcitriol supplementation (comparing Table S2 with Table S3). Similar effects were also observed in the subgroup analysis of age (Tables S4 and S5). When incidence of dementia was compared between two age groups, dementia‐free adults aged 65–75 showed more sensitivity than adults aged 75 or older to vitamin D supplementation (when comparing Table S4 with Table S5).

**TABLE 1:** Baseline characteristics of the matched calcitriol users and nonusers

	Before propensity score match			After propensity score match		
	Users	Nonusers	*p* Value^a^	Users	Nonusers	*p* Value^a^
	*N* = 20108	*N* = 89766		*N* = 7324	*N* = 7324	
Age	74.5 (6.5)	73.9 (6.8)	<.0001	77.0 (5.6)	77.0 (5.6)	1.000
Sex/gender						
Female	13,236 (65.82)	41295 (46.00)	<.0001	4643 (63.39)	4643 (63.39)	1.000
Male	6872 (34,018)	48471 (54.00)		2681 (36.61)	2681 (36.61)	
Osteoporosis						
No	16,733 (83.22)	81035 (90.27)	<.0001	6023 (82.24)	6023 (82.24)	1.000
Yes	3375 (16.78)	8731 (9.73)		1301 (17.76)	1301 (17.76)	
Hyperlipidemia						
No	16,955 (84.32)	83,550 (93.08)	<.0001	6421 (87.67)	6421 (87.67)	1.000
Yes	3153 (15.68)	6216 (6.92)		903 (12.33)	903 (12.33)	
Hypertension						
No	8064 (40.1)	59,677 (66.48)	<.0001	2981 (40.7)	2981 (40.7)	1.000
Yes	12,044 (59.9)	30,089 (33.52)		4343 (59.3)	4343 (59.3)	
Thyroid disorders						
No	19,348 (96.22)	88,916 (99.05)	<.0001	7214 (98.5)	7214 (98.5)	1.000
Yes	760 (3.78)	850 (0.95)		110 (1.5)	110 (1.5)	
Diabetes						
No	14,188 (70.56)	78,234 (87.15)	<.0001	5570 (76.05)	5570 (76.05)	1.000
Yes	5920 (29.44)	11,532 (12.85)		1754 (23.95)	1754 (23.95)	
Renal disease						
No	17,964 (89.34)	88,523 (98.62)	<.0001	7016 (95.79)	7016 (95.79)	1.000
Yes	2144 (10.66)	1243 (1.38)		308 (4.21)	308 (4.21)	
CCI score						
Score 0	9016 (44.84)	47,421 (52.83)	<.0001	3141 (42.89)	3141 (42.89)	1.000
Score 1	5700 (28.35)	17,704 (19.72)		2061 (28.14)	2061 (28.14)	
Score ≥ 2	5392 (26.82)	24,641 (27.45)		2122 (28.97)	2122 (28.97)	
Calcium Prescription						
No	2988	60,062 (66.91)	<.0001	1053 (14.38)	1053 (14.38)	1.000
Yes	17,120	29,704 (33.09)		6271 (85.62)	6271 (85.62)	

*Note*: Age was expressed as mean (SD) and others data were expressed as *n* (%).

^a^ Continuous variables were analyzed using Kruskal–Wallis test, whereas categorical variables (proportions) were analyzed using the chi‐square test.

### 3.5 Vitamin D supplementation is associated with mortality of dementia

**FIGURE 4:**
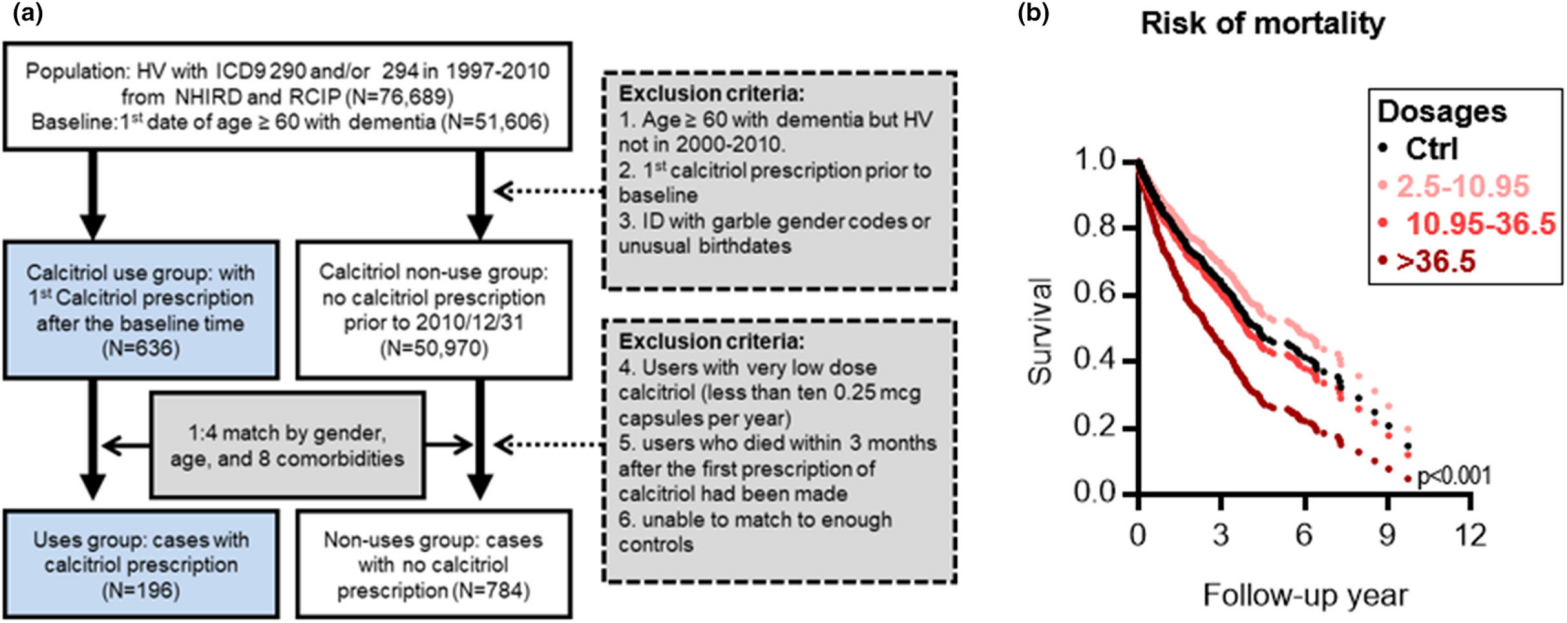
Population‐based cohort study of the associations between risk of mortality and calcitriol use in dementia. (a) Flow chart of cohort formation for epidemiological study of association between survival and calcitriol supplementation in patients with pre‐existing dementia. HV, Registry for catastrophic illness patients; ICD9, International Statistical Classification of Diseases and Related Health Problems (ICD) 9; NHIRD, National Health Insurance Research Database; ID, Registry for beneficiaries; RCIP, Registry of Catastrophic Illness Patients; Comorbidities including calcium prescription, chronic renal disease, osteoporosis, thyroid, parathyroid disorders, hyperlipidemia, hypertension, and Charlson score. (b) The adjusted survival curves among dementia patients with different average dosages of calcitriol. The relationship between mortality and calcitriol use was determined by using the Kaplan–Meier survival curves and log‐rank tests with a follow‐up of up to 10 years (*n* = 980). The model was adjusted for age, sex/gender, calcium prescription, chronic renal disease, osteoporosis, thyroid, parathyroid disorders, hyperlipidemia, and hypertension. ***p < 0.001 by Maximum Likelihood‐ratio test (Ctrl vs. high cumulative doses with >36.5 mcg/year)

Since vitamin D supplementation led to increased Aβ depositions and exacerbated AD led, we wanted to know whether vitamin D supplementation would also increase the risk of mortality in people with pre‐existing dementia. We identified 980 patients diagnosed with dementia who were and were not prescribed calcitriol in the NHIRD and followed them over a 11‐year period (2000–2010) (Figure 4a). They were frequency matched for similar distributions in age, gender, and eight comorbidities (Table 2). The dementia patients prescribed a high cumulative dose of calcitriol (>146 capsules of 0.25 mcg per year) were found to have a 2.17‐fold increase in risk of death, compared with those not prescribed the drug (Figure 4b and Table S6). We found no significant difference in mortality among those taking medium doses (43.8–146 capsules/year) or low cumulative doses (<43.8 capsules/year), compared with nonusers (Figure 4b and Table S6). Similar effects were also observed in the subgroup analysis of sex (Tables S7 and S8). When survival was compared between sex, females with pre‐existing dementia showed worse survival than males with pre‐existing dementia in response to calcitriol supplementation (comparing Table S7 with Table S8). When survival was compared between two age groups, the pre‐existing dementia adults aged 75 and older showed worse survival than the aged 65–75 adults with pre‐existing dementia to calcitriol supplementation (when comparing Table S9 with Table S10). Taken together, these animal model and population‐based results support the conclusion that vitamin D supplementation aggravates the progression of AD.

**TABLE 2:** Baseline characteristics of the matched calcitriol users and nonusers in the survival of dementia cohort

	Users (dosage (mcg/year))			Nonusers	*p* Value^a^
	>36.5 (*n* = 63)	10.95–36.5 (*n* = 64)	2.5–10.95 (*n* = 69)	(*n* = 784)	
Sex/gender					
Female	42 (66.67)	44 (68.75)	47 (68.12)	532 (67.86)	0.9955
Male	21 (33.33)	20 (31.25)	22 (31.88)	252 (32.14)	
Age	80.0 ± 7.0	79.4 ± 6.1	78.9 ± 6.4	79.3 ± 6.3	0.6801
Calcium prescription					
No	18 (28.57)	15 (23.44)	18 (26.09)	204 (26.02)	0.9329
Yes	45 (71.43)	49 (76.56)	51 (73.91)	580 (73.98)	
Renal disease					
No	34 (53.97)	37 (57.81)	47 (68.12)	472 (60.20)	0.395
Yes	29 (46.03)	27 (42.19)	22 (31.88)	312 (39.80)	
Thyroid disorder					
No	61 (96.83)	64 (100.00)	69 (100.00)	776 (98.98)	0.2342
Yes	2 (3.17)	0 (0.00)	0 (0.00)	8 (1.02)	
Osteoporosis/osteopenia					
No	32 (50.79)	30 (46.88)	28 (40.58)	360 (45.92)	0.7012
Yes	31 (49.21)	34 (53.13)	41 (59.42)	424 (54.08)	
Charlson comorbidity score					
Score 0–2	19 (30.16)	16 (25.00)	27 (39.13)	248 (31.63)	0.7173
Score 3–4	21 (33.33)	26 (40.63)	23 (33.33)	280 (35.71)	
Score ≥ 5	23 (36.51)	22 (34.38)	19 (27.54)	256 (32.65)	
Hyperlipidemia					
No	47 (74.60)	43 (67.18)	45 (65.22)	540 (68.89)	0.6869
Yes	16 (25.40)	21 (32.81)	24 (34.78)	244 (31.12)	
Hypertension					
No	13 (20.63)	13 (20.31)	15 (21.74)	164 (20.92)	0.9975
Yes	50 (79.37)	51 (79.69)	54 (78.26)	620 (79.08)	

*Note*: Data were expressed as *n* (%).

^a^ Continuous variables were analyzed using Kruskal–Wallis test, whereas categorical variables (proportions) were analyzed using the chi‐square test.

## 4 DISCUSSES

The level of VDR is usually positively associated with the serum level of 25(OH)D_3_ in healthy adults (Medeiros et al., 2020). Normally, when VDR is inactivated, the serum 1,25(OH)_2_D_3_ levels will be increased to maintain homeostasis. However, a converse relationship between vitamin D concentration and VDR levels can be seen when the 1,25(OH)D_3_‐VDR signaling pathway is impaired during the pathogenesis of chronic diseases. In line with this notion, the findings of this study using an AD mouse model suggest that vitamin D deficiency may be actually more of an early feature or an outcome of AD than a cause of the disease. Other studies report findings that may also support this notion. Older African‐Americans are two to three times more likely to develop AD than elderly whites (Alzheimer's's, 2014; Amadori et al., 2017), while the African‐Americans have higher mean VDR levels (Amadori et al., 2017; Neill et al., 2013; Richards et al., 2017) but much lower serum levels of vitamin D (Dawson‐Hughes, 2004). Another example of the converse relationship between vitamin D concentration and VDR levels has been reported in patients with insulin resistance and obesity, who have been found to have deficient levels of vitamin D on the one hand but increased levels of VDR in adipose tissue on the other (Kang et al., 2015). Future studies may want to explore whether the decrease of vitamin D is actually a common pathological response that occurs in many aging‐associated diseases, because, in addition to AD, patients with vascular disease, thyroid disorders, and osteoporosis are likely to have decreased levels of serum vitamin D and, of course, be at higher risk for dementia (Autier et al., 2014; Duthie et al., 2011).

The findings of our animal experiments also suggest that the prolonged vitamin D supplementation might actually exacerbate AD. To try to understand how vitamin D might have an adverse effect on development and progress of AD, we explored how the VDR pathway might somehow be involved. In our exploration, we found the vitamin D did not rescue the canonical VDR‐RXR pathway but instead further exacerbated the non‐genomic VDR/p53 complex in causing damage to AD brains. The results of our mechanistic analysis are important as we try to clarify why supplementation with vitamin D may not be the best way to address vitamin D deficiency with AD and may not protect older people from dementia.

Importantly, the epidemiological studies that we performed using two nationwide longitudinal cohorts also supported our finding that prolonged supplementation of vitamin D_3_ had adverse effect in AD. We found that long‐term supplementation of vitamin D did not have any benefit on dementia and, it is likely that it increased the risk of dementia in older people and increased mortality in people with dementia. This large‐scale cohort finding is important in that it helps bring into question the wisdom behind the assumption that AD‐associated vitamin D deficiency necessitates need for vitamin D supplementation among older people seeking protection from dementia. These findings, however, do not preclude the potential clinical benefit of vitamin D supplementation on lower AD risk in younger or middle‐aged people before the disease has taken its toll and before AD damage has become irreversible. Supporting this notion, several animal studies have reported that starting vitamin D supplementation at very early stage of disease in AD mice, when amyloid plaques are far from being developed, may benefit AD (Landel, Millet, et al., 2016b; Morello et al., 2018; Wong et al., 2021; Yu et al., 2011). Therefore, timing of supplementation is perhaps an important factor to consider. Accordingly, it may be more prudent to discourage older adults or individuals with dementia from long‐term vitamin D supplementation until large careful clinical trials are performed to prove otherwise.

This cohort study has some limitations. One limitation is that it is a cross‐sectional study and so a causal relationship between long‐term vitamin D supplementation and increased risk of dementia cannot be firmly established. Another limitation of the present study is the lack of generalizability of our findings to other racial and ethnic groups. Finally, the implications of vitamin D deficiency for many other chronic diseases also need to be further investigated by exploring the mechanistic link of initiation of vitamin D deficiency and its causality in those diseases.

Vitamin D is also known the sunshine vitamin because it is synthesized in our skin during exposure to sunlight. This seemingly super nutrient was initially recognized as a key regulator of calcium homeostasis in maintaining bone integrity. Its use has been expanded to cover a wide range of body functions from early life to old age, because a large number of observational studies have demonstrated that a deficiency in vitamin D has been associated with many disorders throughout the body, including not only osteoporosis and fractures but also heart disease, high blood pressure, COVID‐19 infection, immune system disorders, cancer, stroke, and several metabolic disorders (Holick & Chen, 2008; Pereira‐Santos et al., 2015). The hormonal‐like vitamin D exerts a neuroprotective role through genomic action (Brewer et al., 2001). Specifically, vitamin D metabolite 1,25(OH)_2_D_3_ binds to VDR to confer transcriptional activity in the nucleus. However, the results of the current study suggest supplementation of vitamin D may actually worsen brain health in older adults whose blood levels of vitamin D are low. While this finding in surprising, a more interesting research topic would be the mechanism through which vitamin D supplementation worsens the effect of vitamin deficiency. It is possible that the vitamin D‐dependent genomic signaling is impaired in patients with dementia. VDR is also known to act as an important regulator of xenobiotic metabolism in a vitamin D‐independent manner (Krasowski et al., 2011; Reschly & Krasowski, 2006). Thus, it could be that dementia‐related toxic amyloid formation elicits xenobiotic responses through non‐genomic VDR signaling. Supporting this hypothesis, our study using AD mouse model suggests that the non‐genomic VDR/p53 signaling is further activated in AD brains and contributes to neuronal apoptosis. We, therefore, believe the findings of this longitudinal cross‐sectional study, which is supplementation vitamin D exacerbates dementia, may better align with our hypothesis that non‐genomic VDR signaling might contribute to the promotion of dementia.

In conclusion, the results of the current study suggest supplementation of vitamin D may increase the risk of dementia in older adults and increase the risk of mortality in older adults with pre‐existing AD whose blood levels of vitamin D are low. Until our findings are proved otherwise, the use of vitamin D supplementation to prevent dementia should be reconsidered.

## AUTHOR CONTRIBUTIONS

JLJ, RHL, YYH, and MHC involved in conceptualization; RHL, YYH, CCH, BHY, and YRL involved in methodology; RHL, CCH, YYH, JLJ, MHC, and FSS involved in investigation; RHL, CCH, and JLJ involved in visualization; JLJ involved in supervision and writing—review and editing; JLJ and RHL involved in Writing—original draft.

## CONFLICT OF INTEREST

The authors declare no competing interests.

## Supporting information


Appendix S1

## Data Availability

The data that support the findings of this study are available from the corresponding author upon reasonable request.
