# Supplementary material for: Vitamin D supplementation worsens Alzheimer's progression: Animal model and human cohort studies
Source: Aging Cell. 2022 Jul 12;21(8):e13670. doi: 10.1111/acel.13670 (PMC9381901; doi:10.1111/acel.13670)
Supplement: Supplementary file 1 — Appendix S1 [file ACEL-21-e13670-s001.docx]

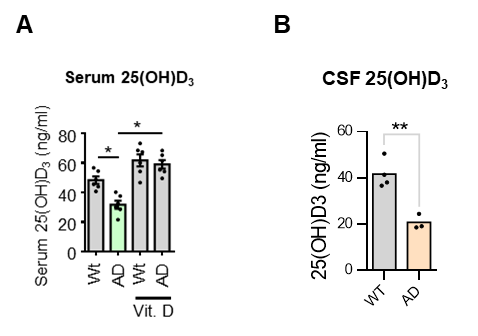
**Supplementary Figure**

**Figure S1.** Cerebrospinal fluid (CSF) 25(OH)D_3_ levels in APP/PS1 mice. 25(OH)D_3_ levels in serum and CSF were determined by Vitamin D3 EIA Kit and the results are shown as mean ± SEM. *P<0.05 by unpaired t-test.

**
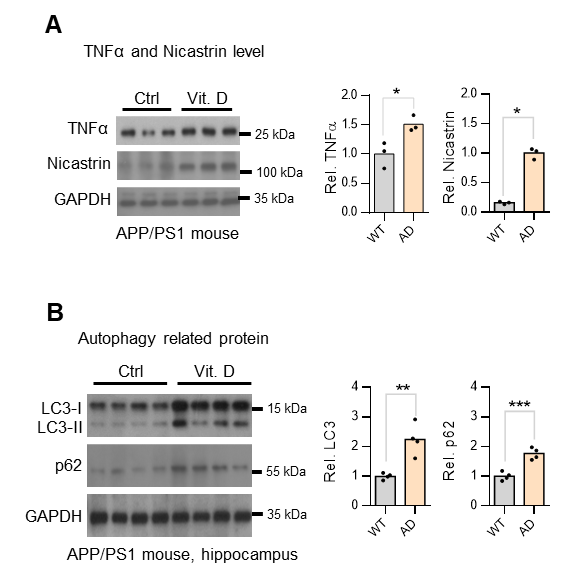
**

**Figure S2. Western blot analysis of inflammatory cytokine and APP processing-related proteins in the hippocampus of APP/PS1 mice supplemented with or without cholecalciferol.** (A) The western blots for hippocampal TNFα, Nicastrin and GAPDH. (B) The western blots for hippocampal LC3, p62 and GAPDH. Densitometrical quantification of target bands were normalized to GAPDH. *P<0.05; **P<0.01; ***P < 0.05 by unpaired t-test (right panel).


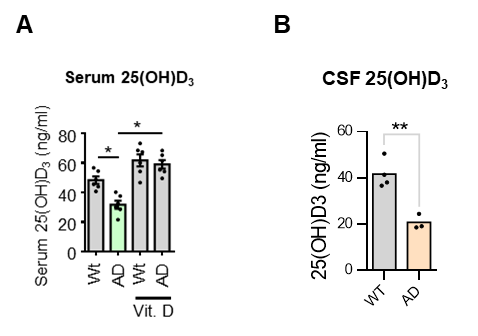


**Figure S3.** Cholecalciferol supplementation restores serum 25(OH)D_3_ levels. 25(OH)D_3_ levels in serum and CSF were determined by Vitamin D3 EIA Kit and the results are shown as mean ± SEM. *P<0.05 by unpaired t-test.

**Table S1. Calcitriol use in people aged over 65 years is associated with increased risk of dementia.**

| **Dosage**  **(mcg/year)^#^** | **Crude HR**  **(95%CI)** | **p-value** | **Adjusted HR**  **(95%CI)** | **p-value** |
| --- | --- | --- | --- | --- |
| **2.5-10.95** | 1.04 (0.91-1.19) | 0.5535 | 1.04 (0.91-1.19) | 0.5833 |
| **10.95-36.5** | 1.26 (1.09-1.46) | 0.0021^*^ | 1.27 (1.09-1.47) | 0.0016^*^ |
| **>36.5** | 1.83 (1.57-2.13) | <.0001^*^ | 1.80 (1.54-2.09) | <.0001^*^ |

# Dosage (mcg/year) is the assumed average maintenance dose per year for a calcitriol used in the whole follow-up. *Significantly different from control group at P < 0.05.

**Table S2**. **Over-supplementation of calcitriol in males aged over 65 years is linked to an increased incidence of dementia (n=5362).**

| **Dosage**  **(mcg/year)^#^** | **Crude HR**  **(95%CI)** | **p-value** | **Adjusted HR**  **(95%CI)** | **p-value** |
| --- | --- | --- | --- | --- |
| **2.5-10.95** | 1.12 (0.89-1.42) | 0.3423 | 1.12 (0.89-1.42) | 0.3314 |
| **10.95-36.5** | 1.14 (0.88-1.48) | 0.3243 | 1.14 (0.88-1.49) | 0.3201 |
| **>36.5** | 1.85 (1.44-2.39) | <.0001 | 1.81 (1.40-2.34) | <.0001* |

# Dosage (mcg/year) is the assumed average maintenance dose per year for a calcitriol used in the whole follow-up. *Significantly different from control group at P < 0.05

**Table S3**. **Over-supplementation of calcitriol in females aged over 65 years is linked to an increased incidence of dementia (n=9286).**

| **Dosage**  **(mcg/year)^#^** | **Crude HR**  **(95%CI)** | **p-value** | **Adjusted HR**  **(95%CI)** | **p-value** |
| --- | --- | --- | --- | --- |
| **2.5-10.95** | 1.00 (0.85-1.18) | 0.9864 | 1.00 (0.84-1.18) | 0.9883 |
| **10.95-36.5** | 1.32 (1.11-1.58) | 0.0020* | 1.33 (1.12-1.59) | 0.0015* |
| **>36.5** | 1.82 (1.51-2.20) | <.0001* | 1.78 (1.47-2.16) | <.0001* |

# Dosage (mcg/year) is the assumed average maintenance dose per year for a calcitriol used in the whole follow-up. *Significantly different from control group at P < 0.05

**Table S4**. **Over-supplementation of calcitriol in people aged 65-75 years is linked to an increased incidence of dementia (n=5196).**

| **Dosage**  **(mcg/year)^#^** | **Crude HR**  **(95%CI)** | **p-value** | **Adjusted HR**  **(95%CI)** | **p-value** |
| --- | --- | --- | --- | --- |
| **2.5-10.95** | 0.95 (0.76-1.20) | 0.6872 | 0.94 (0.75-1.19) | 0.6114 |
| **10.95-36.5** | 1.40 (1.11-1.76) | 0.0048* | 1.41 (1.12-1.78) | 0.0039* |
| **>36.5** | 2.02 (1.56-2.61) | <.0001* | 2.03 (1.57-2.63) | <.0001* |

# Dosage (mcg/year) is the assumed average maintenance dose per year for a calcitriol used in the whole follow-up. *Significantly different from control group at P < 0.05

**Table S5. Over-supplementation of calcitriol in people aged over 75 years is linked to an increased incidence of dementia (n=9452).**

| **Dosage**  **(mcg/year)^#^** | **Crude HR**  **(95%CI)** | **p-value** | **Adjusted HR**  **(95%CI)** | **p-value** |
| --- | --- | --- | --- | --- |
| **2.5-10.95** | 1.10 (0.93-1.31) | 0.2501 | 1.10 (0.93-1.31) | 0.2496 |
| **10.95-36.5** | 1.21 (1.00-1.46) | 0.0481 | 1.20 (0.99-1.45) | 0.0571 |
| **>36.5** | 1.74 (1.44-2.09) | <.0001* | 1.74 (1.45-2.10) | <.0001* |

# Dosage (mcg/year) is the assumed average maintenance dose per year for a calcitriol used in the whole follow-up. *Significantly different from control group at P < 0.05

**Table S6. Calcitriol use is associated with increased risk of mortality in dementia.**

| **Dosage**  **(mcg/year)^#^** | **Crude HR**  **(95%CI)** | **p-value** | **Adjusted HR**  **(95%CI)** | **p-value** |
| --- | --- | --- | --- | --- |
| **<10.95** | 0.79 (0.53-1.18) | 0.2483 | 0.83 (0.55-1.24) | 0.3574 |
| **10.95-36.5** | 1.10 (0.73-1.66) | 0.6370 | 1.17 (0.78-1.76) | 0.4559 |
| **>36.5** | 2.39 (1.64-3.48) | <.0001* | 2.17 (1.48-3.17) | <.0001* |

^#^ Dosage (mcg/year) is the assumed average maintenance dose per year for a calcitriol used in the whole follow-up. *Significantly different from control group at P < 0.05.

**Table S7. Over-supplementation of calcitriol is associated with decreased survival of male patients with pre-existing dementia. (n=315).**

| **Dosage**  **(mcg/year)^#^** | **Crude HR**  **(95%CI)** | **p-value** | **Adjusted HR**  **(95%CI)** | **p-value** |
| --- | --- | --- | --- | --- |
| **<10.95** | 0.67 (0.34-1.33) | 0.2533 | 0.65 (0.33-1.29) | 0.2168 |
| **10.95-36.5** | 1.99 (1.12-3.54) | 0.0195 | 1.97 (1.10-3.54) | 0.0230 |
| **>36.5** | 1.89 (1.02-3.51) | 0.0448 | 2.02 (1.06-3.84) | 0.0330 |

^#^ Dosage (mcg/year) is the assumed average maintenance dose per year for a calcitriol used in the whole follow-up. *Significantly different from control group at P < 0.05.

**Table S8. Over-supplementation of calcitriol is associated with decreased survival of female patients with pre-existing dementia. (n=665).**

| **Dosage**  **(mcg/year)^#^** | **Crude HR**  **(95%CI)** | **p-value** | **Adjusted HR**  **(95%CI)** | **p-value** |
| --- | --- | --- | --- | --- |
| **<10.95** | 0.85 (0.52-1.40) | 0.5180 | 0.94 (0.57-1.55) | 0.8008 |
| **10.95-36.5** | 0.79 (0.44-1.41) | 0.4213 | 0.85 (0.47-1.52) | 0.5764 |
| **>36.5** | 2.67 (1.66-4.29) | <.0001* | 2.50 (1.55-4.05) | 0.0002* |

^#^ Dosage (mcg/year) is the assumed average maintenance dose per year for a calcitriol used in the whole follow-up. *Significantly different from control group at P < 0.05.

**Table S9. Over-supplementation of calcitriol is associated with decreased survival of 60-75 years-old patients with pre-existing dementia. (n=239).**

| **Dosage**  **(mcg/year)^#^** | **Crude HR**  **(95%CI)** | **p-value** | **Adjusted HR**  **(95%CI)** | **p-value** |
| --- | --- | --- | --- | --- |
| **<10.95** | 1.47 (0.70-3.07) | 0.3075 | 1.54 (0.72-3.27) | 0.2657 |
| **10.95-36.5** | 1.30 (0.59-2.85) | 0.5128 | 1.38 (0.59-3.21) | 0.4587 |
| **>36.5** | 1.91 (0.69-5.26) | 0.2134 | 1.35 (0.47-3.87) | 0.5817 |

^#^ Dosage (mcg/year) is the assumed average maintenance dose per year for a calcitriol used in the whole follow-up. *Significantly different from control group at P < 0.05.

**Table S10. Over-supplementation of calcitriol is associated with decreased survival of over 75 years-old patients with pre-existing dementia. (n=741).**

| **Dosage**  **(mcg/year)^#^** | **Crude HR**  **(95%CI)** | **p-value** | **Adjusted HR**  **(95%CI)** | **p-value** |
| --- | --- | --- | --- | --- |
| **<10.95** | 0.63 (0.39-1.02) | 0.0615 | 0.68 (0.42-1.10) | 0.1169 |
| **10.95-36.5** | 1.05 (0.65-1.69) | 0.8545 | 1.08 (0.67-1.74) | 0.7669 |
| **>36.5** | 2.43 (1.62-3.65) | <.0001* | 2.46 (1.63-3.71) | <.0001* |

^#^ Dosage (mcg/year) is the assumed average maintenance dose per year for a calcitriol used in the whole follow-up. *Significantly different from control group at P < 0.05.
